# Enteric populations of *Escherichia coli* are likely to be resistant to phages due to O antigen expression

**DOI:** 10.1128/msphere.00386-26

**Published:** 2026-07-06

**Authors:** Brandon A. Berryhill, Teresa Gil-Gil, Kylie B. Burke, Jake Fontaine, Catherine E. Brink, Mason G. Harvill, David A. Goldberg, Jareth N. Navas, Kerrie L. May, Marcin Grabowicz, Konstantinos T. Konstantinidis, Bruce R. Levin, Michael H. Woodworth

**Affiliations:** 1 Department of Biology, Emory University 1371, Atlanta, Georgia, USA; 2 Department of Medicine, Division of Infectious Diseases, Emory University School of Medicine 12239, Atlanta, Georgia, USA; 3 Ocean Science & Engineering, School of Biological Sciences, Georgia Institute of Technology 1372, Atlanta, Georgia, USA; 4 School of Civil & Environmental Engineering, Georgia Institute of Technology 1372, Atlanta, Georgia, USA; 5 Department of Microbiology and Immunology, Emory University School of Medicine 12239, Atlanta, Georgia, USA; University of Michigan-Ann Arbor, Ann Arbor, Michigan, USA

**Keywords:** *Escherichia coli*, bacteriophage, population biology, phage resistance, fecal microbiota transplantation, enteric microbiome

## Abstract

Metagenomic data provide evidence that bacteriophage (phage) abound in the enteric microbiomes of humans. However, the contribution of these viruses in shaping the bacterial composition of the gut microbiome and how these phages are maintained remain unclear. We performed experiments with 756 combinations of 54 *Escherichia coli* and nine phage isolates from four fecal microbiota transplantation (FMT) doses and five laboratory phages as samples of non-dysbiotic human enteric microbiota. We also developed a mathematical model of the population and evolutionary dynamics of bacteria and phage. Our experiments predict that as a consequence of the production of the O antigen, most of the *E. coli* in the human enteric microbiome will be resistant to infections with the array of co-occurring phages. Our modeling suggests that phages are maintained in these enteric communities due to the high rates of transition between the O antigen-resistant and -sensitive states. Based on our observations and predictions from this theory, we postulate that the phage found in the human gut are likely to play a little role in shaping the strain composition of *E. coli* of healthy individuals. Although we only investigated *E. coli*, the mechanism of resistance described here is shared among most of the gram-negative bacteria. Evidence is provided that, as a consequence of O antigen-mediated resistance, the genetically diverse array of bacteriophage in the gut microbiome of humans plays little or no role in determining the densities and distribution of the genetically diverse strain *E. coli* in this habitat. Our mathematical model predicts and our experiments support the hypothesis that the phage present in the gut microbiome are maintained by replication on the minority of sensitive bacteria generated by the leakiness of O antigen-mediated resistance.

**IMPORTANCE:**

Bacteriophages (phages) are abundant in the human gut, yet whether these viruses shape the bacterial communities living there remains unresolved. Using *Escherichia coli* and phages isolated from the stool of healthy fecal microbiota transplantation (FMT) donors, together with a mathematical model, we show that the vast majority of gut *E. coli* are resistant to co-occurring phages because they express the O antigen, a surface structure that masks the receptors phages use to attach. Despite this widespread resistance, phages persist by replicating on a small, continually regenerated subpopulation of sensitive cells, a phenomenon we term leaky resistance. These findings suggest that phages play a little role in determining which *E. coli* strains dominate the healthy human gut. Because the O antigen is broadly expressed across gram-negative bacteria, this mechanism likely extends well beyond *E. coli* and helps explain why isolating therapeutic phages against many pathogens is difficult.

## INTRODUCTION

The human microbiome is composed of trillions of microbes, including bacteria, viruses, fungi, protozoa, and archaea (1). The composition of the enteric microbiome has been tightly linked to human health and well-being (2–4), as well as many prevalent diseases such as cardiovascular disease, malignancy, malnutrition, and antimicrobial resistance (5–11). Interventions to modify the enteric microbiome may provide new tools to address many of these threats. However, optimal microbiome treatment strategies remain undefined. The answer to these questions requires an understanding of the factors that determine both the composition and diversity of microbes in this habitat. For the enteric microbiome, bioinformatic data have identified not only genetically diverse arrays of *E. coli* but also genetic diversity among the viruses that prey on these bacteria, i.e., their bacteriophages (phages) (12). However, the contribution of these predatory viruses in determining the densities and distribution of species and strains of the bacteria in the human enteric microbiome is largely unknown.

For these phages to play a role in determining the densities and diversity of *E. coli* in the gut microbiome, the dominant populations of *E. coli* in this habitat must be susceptible rather than immune or resistant to the co-existing phage. There are many mechanisms by which bacteria can become immune to phage. In phage immunity, phage adsorb and inject their genetic material into the bacteria but do not replicate; this is distinct from phage resistance. In phage resistance, the bacteria prevent the phage from adsorbing in the first place. There are also several ways by which bacteria can become resistant to phage. The best studied of these mechanisms is envelope resistance, where because of modification of the receptor sites to which the phage adsorb, the bacteria are resistant to infections with these phage (13). Similar to this mechanism—and most relevant for this study—the O antigen component of the lipopolysaccharides (LPS) of gram-negative bacteria can mask the receptor sites of a number of phages, making the bacteria at least partially resistant to phages (other than those that use the O antigen as a receptor) (14–17).

The results of our experiments and analysis of the properties of a mathematical model make two predictions. First, as a consequence of O antigens, the dominant populations of *E. coli* in the human fecal microbiome are resistant to the dominant population of co-existing phages in this habitat. Second, the observed O antigen-mediated resistance is leaky, meaning there is a high rate of transition from the resistant to sensitive states, thereby generating phage-sensitive minority populations of *E. coli* that can maintain the phage in this habitat.

## RESULTS

### A method to isolate phages from fecal samples and test for the sensitivity of co-existing *E. coli* to these phages

**Fig 1:**
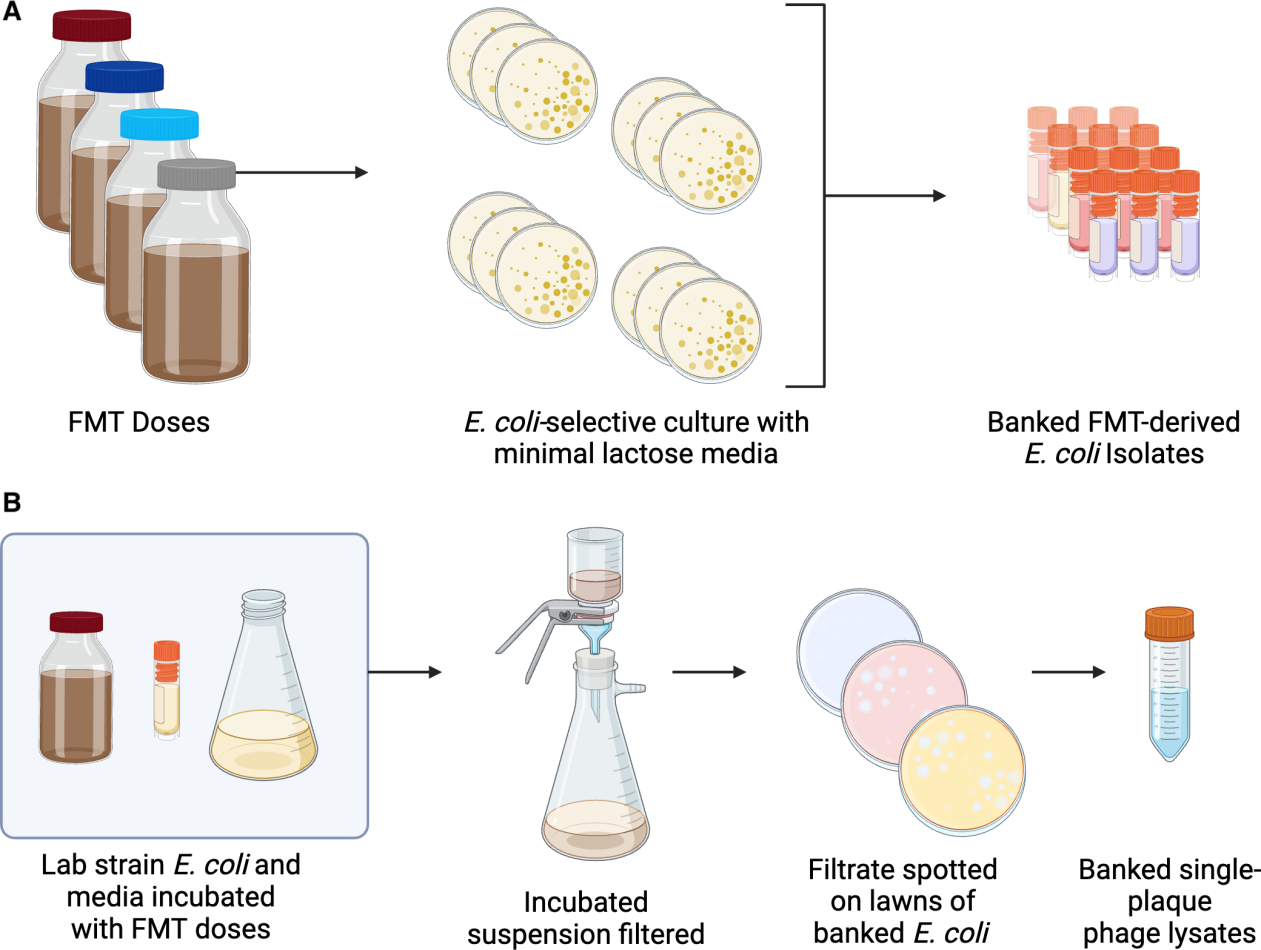
Diagram of bacteria and phage isolation protocol. (**A**) The method employed for isolating the dominant as well as minority populations of *E. coli* from the FMT samples. (**B**) The method employed for isolating bacteriophage from the FMT samples.

To ascertain the susceptibility of bacteria from gut microbiomes of humans to the co-existing phages, we used fecal microbiota transplantation (FMT) doses as sources of enteric *E. coli* from non-dysbiotic hosts. FMT doses are simple suspensions of human donor stool in sterile normal saline, homogenized with a benchtop stomacher device and filtered to exclude large particulate matter. We attempt to isolate phages from these samples in three ways: first, by directly assaying for the presence of phage in these samples by spotting bacteria-free filtrates of these FMT doses onto agar lawns of a broadly phage-sensitive laboratory strain of *E. coli* and searching for zones of inhibition; second, by incubating FMT doses with lysogeny broth (LB) to allow the phage in the FMT doses to replicate on the *E. coli* already present in these samples and then spotting the incubated suspensions onto lawns of a broadly phage-sensitive laboratory strain of *E. coli*; third, by adding both the broadly phage-sensitive laboratory strain of *E. coli* and LB to the FMTs and plating on a laboratory strain of *E. coli*. If *E. coli*-specific phage are present in the sample spotted on the lawns, either clear or turbid zones of inhibition are generated. To obtain cells from minority populations of *E. coli* from these fecal samples, we isolated the bacteria from cultures on *E. coli-*specific (minimal lactose) agar containing different antibiotics (tetracycline; gentamicin; chloramphenicol; ciprofloxacin; ceftriaxone; meropenem; azithromycin; fosfomycin; or colistin). The methods employed to isolate phages and *E. coli* are illustrated in Fig. 1 and described in more detail in Materials and Methods. *E. coli* and phage isolates in these fecal samples underwent whole-genome sequencing, and the diversity and relationships of these bacteria and phage to each other were analyzed with comparative genomic analyses.

### *E. coli* and phage isolated from the FMT doses

We were not able to recover any phage by direct plating of FMT filtrates on lawns of either the laboratory strains or fecal-derived *E. coli*, indicating that the phages in these FMT samples are likely to be of low density, absent, or unable to infect and replicate on the tested *E. coli* strains. We were also not able to isolate phage after incubating the FMT doses with LB, indicating that the *E. coli* in these samples were not able to support the growth of the co-existing phages. We were able to obtain phages from three out of four of the FMTs studied by adding both the broadly phage-sensitive *E. coli* strain and LB in combination*,* indicating that there are low densities of phages in these fecal samples that can replicate on the lab strain of *E. coli*. The phages obtained in this way were not able to replicate on the *E. coli* isolated from these different FMTs, including almost all those from the minority of populations of *E. coli* isolated on the antibiotic plates.

Why were we able to isolate phage by adding both broth and a lab strain of *E*. coli, but not by simply adding broth? One explanation that could explain this observation is that *E. coli* in these fecal samples are already phage-resistant. We hypothesized that these FMT-derived *E. coli* are resistant to the co-existing phage and the laboratory strain is not, due to the expression of the O antigen masking the phage receptor site of these bacteria. Two lines of evidence support this hypothesis. First, these *E. coli* isolated from FMTs bear genes coding for the O antigen. Second, serological tests of these *E. coli* confirm the expression of O antigens (Table 1), with one notable exception. EC1B11 does appear negative by serology, yet it has a predicted functional O antigen cluster. It is known that the sensitivity of this serum assay depends on many factors, and it is likely that this strain has expression of the O antigen below the limit of detection of this assay. Neither the sequence data nor the serological tests demonstrated the existence of the O antigen in the laboratory strains of *E. coli*.

**TABLE 1:** O antigen type and production of FMT-derived *E. coli* isolates

Strain	Predicted O antigen type	Serological confirmation
EC1A16	132	Positive
EC1B11	1	Negative
EC1B13	8	Positive
EC1B14	1	Positive
EC1B22	167	Positive
EC1B24	1	Positive
EC1C2	17/77/44	Positive
EC1C7	17/77/44	Positive
EC1C11	17/77/44	Positive
EC1C12	17/77/44	Positive
EC1C14	17/77/44	Positive
EC1C15	16	Positive
EC1C19	17/77/44	Positive
EC1C20	17/77/44	Positive
EC1C21	17/77/44	Positive
EC1C22	17/77/44	Positive
EC1C16	17/77/44	Positive
EC1C18	17/77/44	Positive
EC1D1	17/77/44	Positive
EC1D2	17/77/44	Positive
EC1D4	17/77/44	Positive
EC1D6	74	Positive
EC1D7	74	Positive
EC1D8	17/77/44	Positive
EC1D9	17/77/44	Positive
EC1D12	74	Positive
EC1D13	74	Positive
EC1D14	74	Positive
EC1D15	17/77/44	Positive
EC1D17	17/77/44	Positive
EC1D18	17/77/44	Positive
EC1D19	17/77/44	Positive
EC1D10	17/77/44	Positive
EC1D11	74	Positive

### The expression of the O antigen makes *E. coli* resistant to phage

To test the hypothesis that O antigen production by *E. coli* mediates resistance, we performed spot assays with MG1655 (an O antigen-negative lab strain), MG1655L5 (an isogenic mutant of MG1655 producing a medium level of the O antigen), and MG1655L9 (an isogenic mutant of MG1655 strongly producing the O antigen), and the phage T7. For further details on the bacterial strains and their sources, see Materials and Methods. We have restricted the assays with our constructed O antigen strains to phage T7 for two reasons: i) this phage is known to have its receptor masked via steric inhibition due to the production and display of the O antigen and ii) bacteria require two genomic mutations to generate classical receptor site resistance to this phage, making the evolution of resistance unlikely in our assays (18). When phage are spotted onto lawns of MG1655L9, no plaques are observed. This observation is parallel to the corresponding experiments with the *E. coli* strains isolated from FMT doses. Lawns of MG1655L5 do support the production of plaques, but those plaques are turbid rather than clear as they are on MG1655. We interpret the turbidity of the plaques found with the highly lytic phage T7 and MG1655L5 as support for the proposition of intermediate phage susceptibility.

### Accounting for the presence of phage in the apparently resistant populations of *E. coli*.

**Fig 2:**
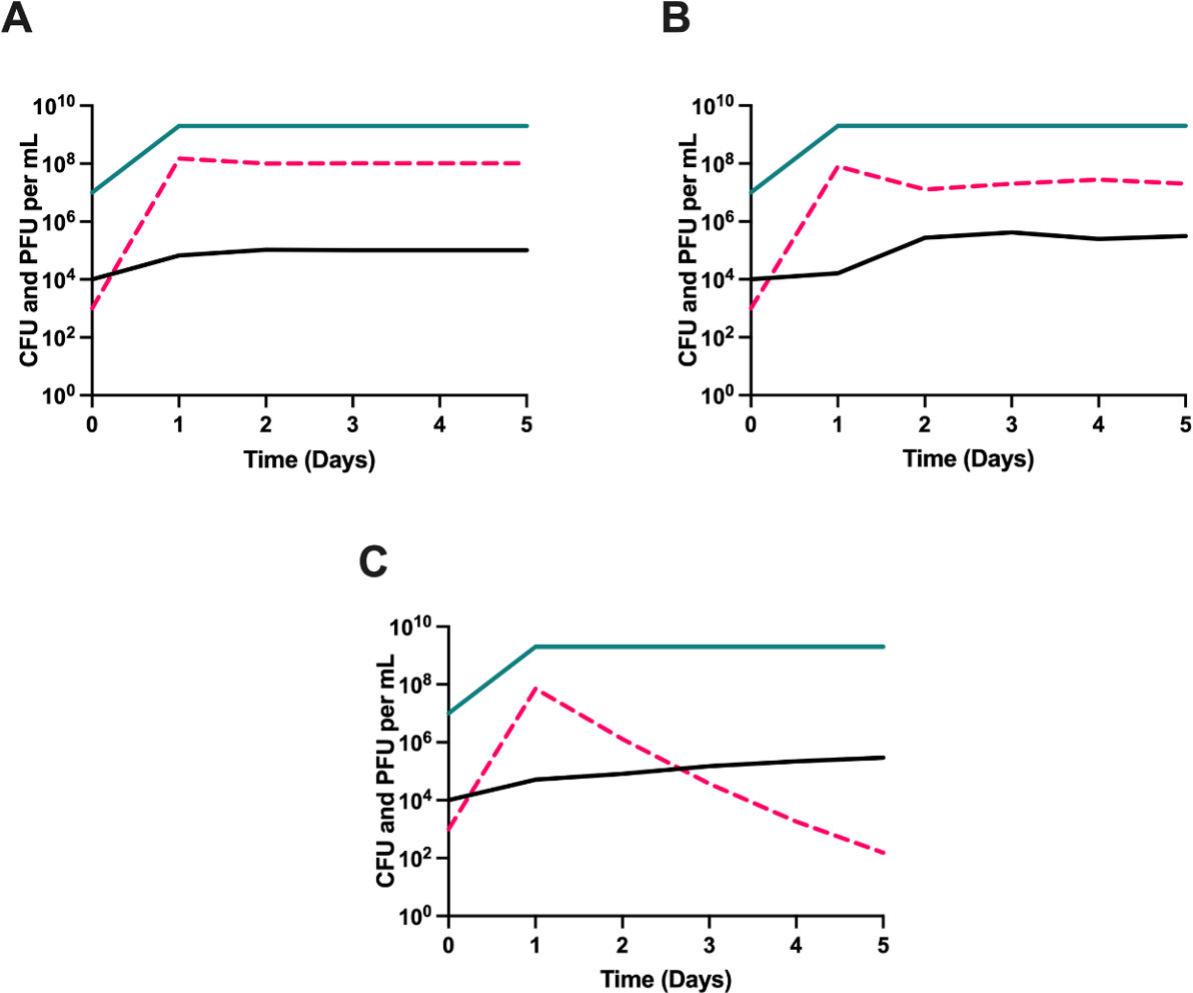
Predicted serial transfer dynamics of O antigen-mediated resistance and a lytic phage. Simulation results showing the densities of bacteria and phage in serial transfer culture with three different transition rates between the resistant and sensitive states. At each simulated transfer, medium containing 1,000 µg/mL of the limiting resource is added along with 0.01 mL of the culture at 24 h after the previous transfer. We assume no fitness cost to resistance, and the transition rate in both directions is the same. (**A**) Transition rates from sensitive to resistant and from resistant to sensitive at 10^−3^ per cell per hour. (**B**) Transition rates from sensitive to resistant and from resistant to sensitive at 10^−4^ per cell per hour. (**C**) Transition rates from sensitive to resistant and from resistant to sensitive at 10^−5^ per cell per hour. O antigen-expressing cells shown in green, sensitive cells shown in black, and phage shown in pink broken lines.

If the dominant populations of *E. coli* in these fecal samples are resistant to the co-existing phage, how are the phage isolated from these samples maintained in the host enteric microbiota? Our lytic phage model (described in Fig. S1 and within the supplemental text, “Model of the population dynamics of resistant O antigen bearing bacteria and lytic phage” section) illustrates that if the rate of transition from the O antigen-resistant state to the sensitive state is sufficiently high (10^−4^ per cell per hour or higher), the phage will be maintained by growing on the minority sensitive population, the predictions of which are described in Fig. 2.

The model described above assumes no fitness cost for resistance and assumes that the transition rate is the same whether bacteria are transitioning to sensitive or resistant. In Fig. S2, we consider the effects of varying fitness costs and unequal transition rates between sensitive and resistant and find their impact to be negligible. We further explore in Fig. S3 the effects of using a temperate phage, with the model described in the supplemental text, “Model of the population dynamics of resistant O antigen bearing bacteria and temperate phage” section. While the dynamics do differ slightly, the maintenance of free phage can still be explained by leaky resistance.

**Fig 3:**
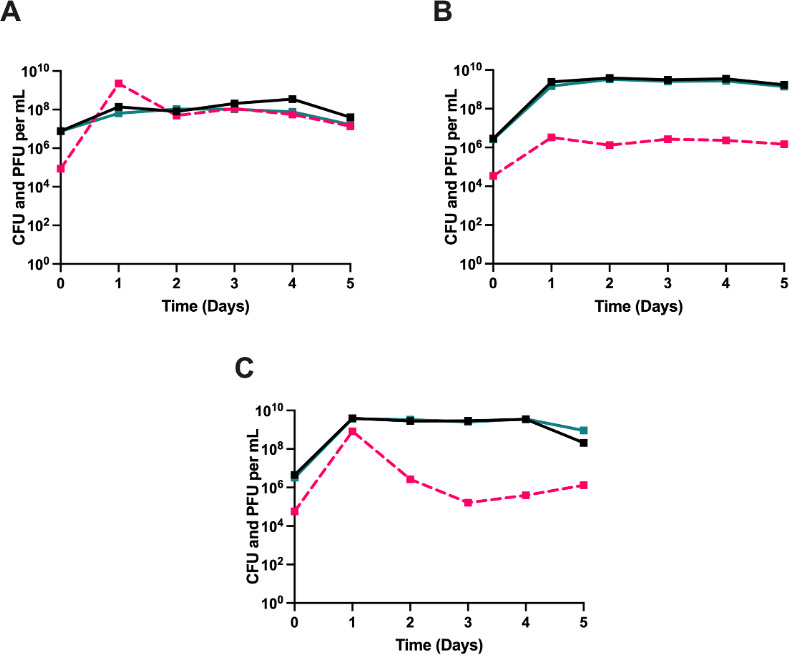
Serial transfer with phage T7 and strains of *E. coli* MG1655 lacking and expressing different levels of the O antigen. Three variants of *E. coli* MG1655 were serially transferred for 5 days in LB with the phage T7. Bacterial densities are shown in solid lines, with the black line representing a phage-free control. The broken pink lines represent phage densities. The turquoise line represents the bacterial density in the presence of the phage. One of three biological replicates is shown. (**A**) Wild-type MG1655 that does not produce the O antigen. (**B**) MG1655L5, which produces a medium amount of the O antigen. (**C**) MG1655L9, which produces a high amount of the O antigen. Two other, qualitatively similar, replicates are presented in Fig. S5. These results show lower but consistently measurable phage densities in *E. coli* populations expressing the O antigen.

To empirically test this leaky resistance hypothesis, we performed serial transfer experiments with the phage T7, MG1655, MG1655L5, and MG1655L9. The results of these experiments are presented in Fig. 3. In serial transfer culture, all conditions (O antigen negative, low levels of O antigen, and high levels of O antigen) were able to maintain the phage. However, phage densities were significantly lower in O antigen-expressing strains compared to wild-type MG1655. A mixed-effects linear regression model showed a significant interaction of strain and serial transfer day (*P* = 0.003) and pairwise differences in mean plaque-forming unit (PFU) density for wild-type MG1655 vs L5 (0.008) and wild-type vs. L9 (*P* = 0.011), consistent with O antigen-mediated resistance reducing but not eliminating phage replication. The predictions of our model, along with these experimental results, explain how phage can be maintained in populations of bacteria resistant to these viruses. This accounts for how we were only able to recover phages after adding an O antigen-negative strain of *E. coli* and LB. It is known that loss of the O antigen corresponds with an increase in vancomycin resistance in *E. coli* (19); therefore, to estimate the frequency of spontaneous O antigen lost due to leaky resistance, we estimate the frequency of vancomycin-resistant subpopulations in otherwise isogenic O antigen-expressing MG1655L9. We find that the rate of spontaneous O antigen loss is 7.08 × 10^−6^ ± 6.66 × 10^−6^. Furthermore, to ensure the serial transfer results are not specific to the O antigen serotype presented in Fig. 3, we conducted parallel serial transfer experiments with one of each of the serotypes in Table 1. Parallel results were obtained and are shown in Fig. S4.

### Genomic diversity of *E. coli* and its phages in FMT doses

**Fig 4:**
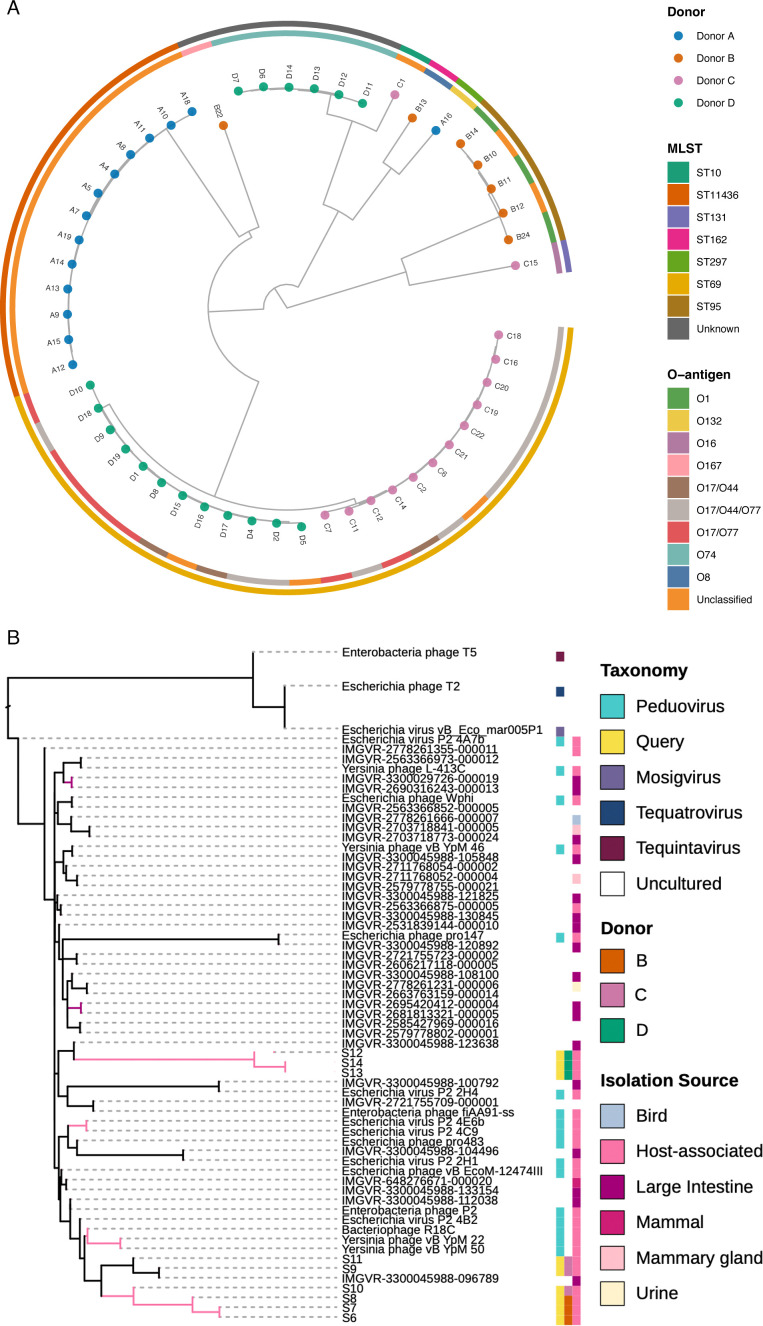
Diversity of *E. coli* and phages isolated from FMT doses. (**A**) Neighbor-joining tree constructed from pairwise average nucleotide identity (ANI) of 53 *E. coli* genomes cultured from four healthy stool donors. Tip nodes are colored by donor; inner and outer rings indicate *in silico-*predicted O-antigen serotype and multilocus sequence analysis sequence type (MLST), respectively. Each donor harbored multiple distinct lineages differing in ANI, MLST, and predicted O-antigen. (**B**) Bacteriophage consensus maximum-likelihood phylogenetic tree based on the core genes of the phage genomes in this study and close relatives from public databases. Thirty-two genes were shared in more than 55/60 of the genomes in the major AAI cluster of Fig. S6 and were used to build individual trees, as described in Materials and Methods. Phage characteristics are indicated by color strips adjacent to the phage tree, from left to right: Phage taxonomy for known sequences, donor in this study from which phage was isolated, and isolation source of phage genome from public databases.

We identified an apparent dominant and several minority *E. coli* strains in each FMT dose. These results demonstrate successful isolation of distinct populations of *E. coli* in the fecal microbiomes of several healthy donors. For each FMT donor, we assessed the diversity of recovered *E. coli* strains via alignment of predicted gene clusters, average nucleotide identity (ANI), *in silico* multi-locus sequence analysis (MLSA), and *in silico* O serotype prediction; we display these results in Fig. 4A. Quality statistics for reads and assemblies are summarized in File S1. We chose to predict the O serotype *in silico* as the diverse expression of these antigens was traditionally a measure of *E. coli* diversity (20). When applying an ANI-based genomovar definition of >99.5% ANI, we identified a median of 2.5 (range 2–3) culturable *E. coli* strains per donor (21). In general, classification by multi-locus sequence typing (MLST) was consistent with the ANI-based clustering. However, strains with similar ANI and of the same MLST displayed a greater diversity in the predicted O antigen type. These findings mirror those of previous studies (22), which found that healthy individuals carry diverse *E. coli* strains, confirming that our methods are readily capable of capturing the diversity in the fecal microbiome.

Of the four FMT donors, phages were recovered from three donors (Donors B, C, and D). Three unique phages were recovered from each of these three donors; thus, we recovered a total of nine distinct phages. Of note, we were not able to recover any phages from Donor A with any of the above-described isolation methods. A phylogenetic analysis of the phage genomes was conducted by generating an average amino acid identity (AAI) heatmap (Fig. S6) and consensus maximum-likelihood phylogenetic trees based on the core phage genomes (Fig. 4B). The reference *Peduovirus* genomes, genomes isolated in this study, and the IMG/VR (Integrated Microbial Genomes/Viruses) genomes shared AAI values of 90%, which reflects the high similarity in gene content between these phages. The consensus tree of these gene trees, estimated using Astral (23), is shown in Fig. 4B. The phylogeny demonstrates that the genomes of Donor D are only distantly related to those of B and C. The phages from Donors B and C also appear to cluster separately, with the exception of a single phage, which is closely related to the phage genomes of Donor B despite being isolated from Donor C. This suggests that each FMT donor has multiple distinct culturable phage populations and that the populations within each donor are largely distinct from the other donors.

### Susceptibility of *E. coli* isolates to phage

Our results indicate that while a high level of expression of the O antigen is sufficient to allow phage resistance, phages are still able to be maintained by that resistant population. Furthermore, the diverse *E. coli* isolated from the FMTs were found to display a variety of O antigen types. These observations, taken together, suggest that *E. coli* isolated from healthy human enteric microbiota are likely to be resistant to a diversity of *E. coli* phages, including those from the same community.

To get a better idea of the phage susceptibility/resistance of the *E. coli* in the FMTs, we spot-tested three classes of phage on lawns of each of the genetically distinct bacteria isolated from the FMTs: (i) five well-characterized laboratory phages not isolated from feces; (ii) the co-existing phages isolated from the same donor; and (iii) phages isolated from the other donors. In total, we tested 756 combinations of 54 bacterial isolates and 14 phage isolates.

**Fig 5:**
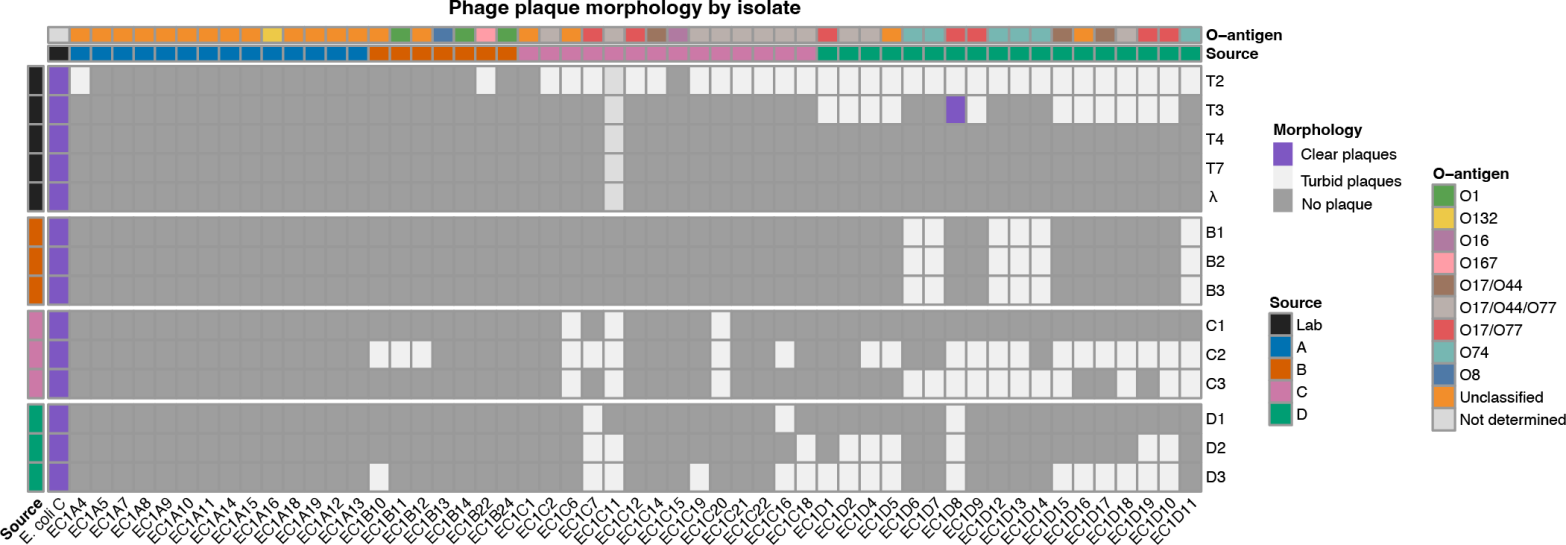
FMT-isolated *E. coli* do not give clear plaques when spotted with laboratory or FMT-derived bacteriophages. Well-characterized laboratory and FMT-derived *E. coli* strains (indicated by source as lab or donor A through D) were spotted with well-characterized laboratory phages (T2, T3, T4, T7, and a virulent mutant of phage λ) and FMT-derived bacteriophages (isolated from FMT donors B through D). Dark blue denotes no plaque formation, light gray denotes turbid plaque formation, and orange denotes clear plaque formation. Clear plaques were almost exclusively produced by the tested phages on lawns of a laboratory strain of *E. coli* C, indicating broad resistance or immunity of FMT-derived *E. coli* to phages from all sources.

Lawns of the laboratory strain *E. coli* C lab displayed clear plaques in spot tests with the 14 tested lab and FMT-derived phages (Fig. 5). This is not the case for the five lab phages tested on lawns of the 53 FMT-derived *E. coli* isolates, which give either no plaques (217/265, 81.9%) or turbid plaques (42/265, 15.8%). There is one exception: the phage T3 spotted on EC1D8 generated a clear plaque (0.3%). When testing the nine FMT-derived phages on lawns of the 53 FMT-derived *E. coli* isolates, 392/477 (82.2%) produced no plaques and 85/477 (17.8%) produced turbid plaques.

For phages in the FMT doses to shape the composition of a recipient’s enteric microbiota, the recipient’s *E. coli* must be sensitive to FMT-derived phage. However, when comparing combinations of FMT-derived phage and *E. coli* isolated from the same or different donors, we do not find sensitivity. We found a significantly higher proportion of turbid plaques with autologous (bacteria and phage from the same donor) phage-*E. coli* combinations compared to allogeneic (bacteria and phage from different donors) phage-*E. coli* combinations (29/117, 24.5% vs 56/360, 15.6%; chi-square *P*-value 0.033). Further analysis of these and the above pairs of bacteria and phage is described in Table S1. These results, taken together, indicate that FMT-derived *E. coli* are less sensitive to phages than laboratory *E. coli* strains—not just FMT-derived phages, but phages in general. To determine if the O antigen is sufficient to explain this generalized pattern of resistance, we obtain O antigen-negative mutants of each serotype (Table 1) and assess their phage susceptibility (Table S2). Importantly, loss of the O antigen broadly restored phage susceptibility.

Interestingly, the expression of the O antigen in the population is not merely coincidental. We find that the O antigen is selected for in the presence of phage, as shown in Fig. S7. In this figure, a low density of O antigen-expressing cells increases in frequency when rare within a population of O antigen cells when exposed to the phage. In the presence of phage T7, O-antigen-expressing cells (MG1655L9) increased from ~ 0.008% to 98.1% of the population within 24 h, compared to 0.09% without phage (*t*-test *P* = 2.9 × 10⁻⁵). Could phage be one of the many selective pressures responsible for the ubiquity of the O antigen in natural populations of *Enterobacteriaceae*?

## DISCUSSION

We set out to elucidate the role of bacteriophages in shaping the composition of *E. coli* in the enteric microbiome of healthy humans. Our interpretation of these results is that bacteriophages likely play a little role in determining the strain composition of co-occurring *E. coli* due to the high prevalence of phage resistance in the enteric microbiome of healthy individuals. In the case of *E. coli*, the expression of the O antigen is sufficient to confer phage resistance while allowing for the maintenance of phage due to the random transition between a phage-resistant and -sensitive state, which we call leaky resistance. Several diverse mechanisms can convey bacteriophage resistance (such as receptor site mutation and the carriage of phage resistance genes on prophage (24, 25)), but we demonstrate that the expression of the O antigen is sufficient to account for the resistance of *E. coli* to a broad swath of phages, including those to which they have never been exposed. Our models and experiments support the hypothesis that despite this resistance, phage will be maintained in communities of these O antigen-expressing bacteria. Moreover, we expect that this same resistance could be observed in many natural populations of *Enterobacteriaceae* as this family broadly expresses the O antigen. Stated another way, we postulate that resistance to co-occurring phages will be the dominant characteristic of bacteria in stable natural communities (26). This prediction is consistent with experimental studies of lytic and temperate bacteriophages *in vitro* (18, 27).

Resistant mutants almost always emerge and ascend to become the dominant population of bacteria in *in vitro* experiments with laboratory strains of bacteria and lytic phages. Nevertheless, phages are commonly maintained in these cultured populations that are dominated by resistant bacteria (27, 28). There are a variety of mechanisms by which the phage can be maintained, but their maintenance appears to play a little role in regulating the densities of these bacterial cultures, and these bacterial populations are more likely shaped by other factors such as resource availability. We postulate this could also be the case in the gut microbiome. While there are many other mechanisms by which phages can be maintained, such as physical refugia as a consequence of the structure of the gut or spontaneous temperate phage induction (Fig. S3), we have demonstrated that leaky resistance is sufficient to independently account for the results observed here. There is, however, a caveat to our purported mechanism for phage maintenance, in that one prediction of our model is that the density of bacteria would exceed that of phage in natural populations. How common that is in natural communities is unclear, as in marine settings, it is thought that the density of phage far exceeds that of the bacteria (29, 30), though recent studies have questioned how high this ratio actually is (31). There is another caveat, in that we have restricted our study to human samples because of the clinical relevance. Other studies by colleagues have examined the phage and *E. coli* interactions in the microbiome of other animals, such as horses (32). Despite these caveats, we believe the mechanism proposed here happens not only in the gut but more broadly in nature. We cannot say definitively how pervasive these observations are, but this question of generality could be answered by other investigators with culture-independent approaches.

We expect that the approach developed in this study also has broader utility for translational phage investigation. If phages are to be developed as therapeutics or as components of therapeutic consortia, we expect that this will require complementary culture-based and culture-independent techniques that include experimental isolation, characterization, and strain-resolved analysis in the context of complex communities.

Interestingly, the mechanism of resistance explored here, the O antigen, is known to be the target of some bacteriophages (33). If the results presented here for *E. coli* are supported in other members of *Enterobacteriaceae*, we would expect difficulties in isolating phage capable of lysing specific bacterial isolates. This anticipated result is echoed by investigators working on isolating phage for therapy, where the rates of finding phage for an isolate are less than 50% for some pathogens (34–36). Here, we provide one explanation for why this could be the case: that is, resistance associated with production of the O antigen.

## MATERIALS AND METHODS

### Fecal microbiota transplant doses

Healthy human participants were recruited and provided informed consent to participate in a stool donor protocol approved by the Emory University Institutional Review Board (Protocol 00112302, PI Woodworth). Donors completed health and behavior questionnaires and blood, urine, and stool laboratory testing to screen for potential pathogens, as described in the PREMIX trial (37). Each stool specimen from a single donor was divided by weight, with 50–100 g of stool in a Seward filter bag and suspended in 250 mL USP sterile saline using a Seward benchtop stomacher at 200 rpm for 30 s twice. After removing the filter bag, the remaining suspension of stool in saline was transferred back to the 250-mL saline bottle and delivered fresh without freezing for *E. coli* and phage isolation, as described below. Additional aliquots with and without 10% glycerol by starting volume were prepared and stored at −80°C.

### Bacterial strains

*E. coli* C was obtained from Marie-Agnès Petit at INRAE in Joy-en-Josas, France. MG1655 L9 and L5 were obtained from Douglas Browning at Aston University in Birmingham, UK (38). Briefly, these constructs were made by cloning either the *rfb* cluster or *wwbL* into isogenic MG1655. Wild *E. coli* were isolated from fecal microbiota transplantation doses, as described below.

### Bacteriophages

Phages Lambda^VIR^, T2, T3, T4, and T7 were acquired from the Levin Lab’s phage collection. Wild phages were isolated from fecal microbiota transplantation doses, as described below.

### Growth media and conditions

Unless otherwise noted, all bacteria were cultured in lysogeny broth (LB) at 37°C, prepared according to the manufacturer’s instructions (Difco, 244620). LB soft agar was made with 0.7% wt/vol agarose, and LB plates were made with 1.6% agarose. LB phage plates were prepared as LB plates supplemented with 20 mM CaCl_2_.

### Isolating bacteria from the FMTs

Serial dilutions of FMT in 0.85% NaCl solution were followed by plating on Davis Minimal (DM) plates (Sigma-Aldrich, 15758) supplemented with 0.4% wt/vol lactose (Sigma-Aldrich, L3750). To isolate unique subpopulations of *E. coli*, isolation was done on minimal lactose plates containing varying antibiotics at the breakpoint concentration (see Fig. 5). Twenty individual colonies were randomly chosen and picked with a sterile stick and simultaneously streaked onto an EMB plate and a DM plate. Both were incubated at 37°C overnight. Isolates colored green on the EMB plate were picked from their respective minimal plate and serially streaked from the DM lactose plate onto another DM lactose plate and incubated at 37°C overnight. Each isolate was labeled, stored, and frozen.

### Isolating phage from the FMTs

FMT suspension (0.1 mL) was inoculated into 10 mL LB broth with ~1e^7^ CFU/mL *E. coli* C. These flasks were grown with shaking at 37°C overnight. The next day, samples were centrifuged and filtered through a 0.22-micron filter (Basix, 13-1001-06). These rough lysates were then plated for single plaques. Individual plaques were picked with a sterile stick and resuspended, as described above, to obtain clonal phage lysates.

### AAI heatmap

We identified the closest relatives to the genomes of this study (query genomes) using whole-genome searches against public genome databases. The final set of genomes used in subsequent analyses consisted of 98 genomes—including the nine query genomes, 34 genomes from the IMG/VR v4 database (39), and 55 genomes that are publicly available on NCBI. Prodigal v2.6.3 (40) was used for gene prediction with default settings, and pairwise all-versus-all average amino acid identity (AAI) was calculated using the aai.rb script from the Enveomics collection (41), with default settings except for decreasing the number of minimum hits to 20. The final heatmap was constructed using the pheatmap package in R. Accession numbers of the public genomes are labeled in the figure.

### Core gene tree

The subset of genomes that make up the major cluster in the AAI heatmap were used for gene clustering at 30% identity with MMseqs2 v 13.4511 (42). The resulting clusters were used to identify the core genome—the genes that are found in 55/60 (~90%) of the genomes. The 32 core genes were extracted using seqtk-1.4, and individual gene alignments were generated using MUSCLE v3.8.31 (43) with default settings. Each alignment was used to generate a maximum-likelihood gene tree in MEGA11 (43) with default parameters. These gene trees were concatenated and passed into Astral 5.7.8 (23) to generate the final consensus tree as Astral has a built-in algorithm to deal with any missing data in the input gene trees. The final tree was visualized and annotated in iTol (44) and is shown in Fig. 4B. The tree was rooted using *Escherichia virus vB_eco_mar005P1*. The additional two genomes used as outgroups to root this tree were added manually.

### Phage susceptibility testing

Approximately 1e^6^ of each phage lysate was spotted onto lawns of the bacteria using the double-layer soft agar technique, as described in reference 45. Phage lysates were diluted 1:100 prior to spotting to minimize lysis from without.

### Sampling bacterial and phage densities

Bacteria and phage densities were estimated by serial dilutions in 0.85% NaCl solution followed by plating. The total density of bacteria was estimated on LB (1.6%) agar plates. To estimate the densities of free phage, chloroform was added to suspensions before serial dilutions and double-layer soft agar enumeration.

### Serial transfer experiments

All serial transfer experiments were carried out in 10 mL LB broth cultures grown at 37°C with vigorous shaking. The cultures were initiated by 1:100 dilution from 10-mL overnight cultures grown from single colonies. Phage was added to these cultures to reach the initial density of ∼10^7^ PFU/mL. At the end of each transfer, 0.1 mL of each culture was transferred into flasks with fresh medium (1:100 dilution). Simultaneously, 0.1-mL samples were taken for estimating the densities of CFUs and PFUs, as described above.

### Estimation of the rate of spontaneous O antigen loss

Serial dilutions of MG1655L9 were plated on LB plates with 175 μg/mL of vancomycin. One hundred surviving colonies from each replicate were screened for sensitivity to the phage P1—sensitivity to which indicates a loss of the O antigen.

### Phage susceptibility due to O antigen loss in FMT-derived *E. coli*

Serial dilutions of the FMT-derived *E. coli* were plated on LB plates containing 3× the ancestral MIC of vancomycin. Surviving colonies from each replicate were screened for sensitivity to the phage P1—sensitivity to which indicates a loss of the O antigen—as well as T2, T4, and T7.

### Antibiotics and their sources

Streptomycin (S6501) was obtained from Sigma-Aldrich; tetracycline (T1700) from Research Products International; gentamicin (BP918-1) from Fisher BioReagents; chloramphenicol (C5793) from Sigma-Aldrich; ciprofloxacin (A4556) from AppliChem; ceftriaxone (C5793) from Sigma-Aldrich; meropenem (QH-8889) from Combi-Blocks; azithromycin (3771) from Tocris; fosfomycin (P5396) from Sigma-Aldrich; and colistin (C4461) from Sigma-Aldrich. All antibiotic Sensi-Discs were obtained from Becton Dickinson.

### Invasion when rare experiment

*E. coli* MG1655 and *E. coli* MG1655L9 were co-cultured in shaking flasks at 37°C at a ratio of 1:1,000 (10^3^:10^7^ CFU/mL), either with or without phage T7 (10^7^ PFU/mL). Bacterial and phage densities were estimated at 0 and 24 h.

### O antigen antiserum assay

*E. coli* O antigen-specific antiserum was obtained from SSI Diagnostica and used as detailed in their protocol for the slide agglutination assay. Each strain was tested with the O antisera, which corresponded to its predicted O antigen type, to confirm the expression of this gene cluster. Slide agglutination was performed to identify O antigens. Strains were grown overnight at 35°C–37°C on MH agar media. A small drop (~20 µL) of specific antiserum was placed on a glass slide and inoculated with 3–5 colonies using an inoculating loop to achieve a distinct milky turbidity. The slide was tilted for 5–10 s and examined under indirect illumination against a black background. A positive reaction was recorded if visible agglutination occurred within 10 s. Reactions occurring after this window or those maintaining a homogenous milky turbidity were considered negative. Individual colonies from positive tests were further subcultured to ensure purity for subsequent analyses. A known O antigen-negative lab strain (MG1655) and its construct bearing the O antigen (MG1655L9) were used as negative and positive controls, respectively.

### Whole-genome sequencing

Samples were sent to MIGS (Pittsburgh, USA) as bacterial colonies grown on an agar plate or as sterile phage lysates for extraction and Illumina sequencing. Individual colonies and lysates were extracted as per the manufacturer’s protocol using the Zymo DNA miniprep bead-beating lysis kit. Sample libraries were prepared for Illumina sequencing using Illumina’s DNA Prep kit and IDT 10-bp unique dual indices and sequenced on an Illumina NovaSeq 6000, producing paired-end 151-bp reads. Demultiplexing, quality control, and adapter trimming were performed with bcl-convert (v4.1.5). Illumina reads were quality-filtered using Trimmomatic (46). *E. coli* C host sequences were depleted from phage lysate sequencing data by mapping to a closed *E. coli* C genome with bowtie2 (47). After trimming and host decontamination (for phage lysates), remaining reads were assembled *de novo* using SPAdes v3.13 (48). Pairwise comparisons of average nucleotide identity on the assembled genomes were performed with the Mashmap method using fastANI v1.32, as in reference 49. Gene sequences were predicted with Prodigal v2.6.3 (40) and annotated with Prokka v1.14.6 (50). O and H antigens were predicted *in silico* with Ectyper v2.0.1 (51). Multilocus sequence analysis (MLSA) typing was performed using mlst v2.23 (Seemann, https://github.com/tseemann/mlst) with the Achtman seven-locus scheme and the PubMLST database (52). ANI values were converted to pairwise distances (100 − ANI) and used to construct a neighbor-joining phylogenetic tree with the ape package v5.7 in R v4.3 (53). The tree was midpoint-rooted using phytools v2.0 (54), and negative branch lengths were set to a minimum of 0.0001. The tree was visualized as a circular phylogram using ggtree v3.10 and ggtreeExtra v1.12 (55, 56), with concentric annotation rings indicating donor identity, predicted O-antigen serotype, and MLSA sequence type.

### Numerical solutions (simulations)

For our numerical analysis of the coupled, ordered differential equations presented in the Supplemental Text, we used Berkeley Madonna with the parameters presented in Table S3. Copies of the Berkeley Madonna programs used for these simulations are available at https://www.eclf.net/.

### Statistical analysis

Statistical tests of difference in proportions of phage plaquing results tabulated as a contingency table were performed with the chisq.test function in the stats package version 4.3.0 in R version 4.3.0 using the RStudio interface version 2023.06.0. Differences in phage densities across host strains in serial transfer experiments were assessed using a linear mixed-effects model fit by restricted maximum likelihood (REML) with the lme function in the nlme package, with log₁₀-transformed PFU/mL as the response, strain, transfer day, and their interaction as fixed effects, and biological replicate as a random intercept. *Post hoc* pairwise comparisons between strains were performed on estimated marginal means with Bonferroni correction for multiple comparisons. Differences in bacterial densities and frequencies between conditions in the invasion when rare experiment (Fig. S7) were assessed using two-sample *t*-tests on log₁₀-transformed densities. Resulting *P*-values < 0.05 were considered statistically significant.

## Data Availability

The Berkeley Madonna program used for the simulations is available at https://www.eclf.net. All raw sequence data for the *E. coli* and phage genomes have been deposited in the NCBI Sequence Read Archive (SRA) and can be found under BioProject accession number PRJNA1028583.

## AFTER EPUB

*[This article was published on 6 July 2026 with a misspelling of Jareth N. Navas's given name and without Kerrie L. May in the byline. The byline was corrected in the current version, posted on 13 July 2026.]*

## SUPPLEMENTAL MATERIAL

File S1 msphere.00386-26-s0001.csv *E. coli* genome quality statistics.

Supplemental material msphere.00386-26-s0002.docx Supplemental text, Fig. S1 to S8, Tables S1 to S3, and File S1 caption.

ASM does not own the copyrights to Supplemental Material that may be linked to, or accessed through, an article. The authors have granted ASM a non-exclusive, world-wide license to publish the Supplemental Material files. Please contact the corresponding author directly for reuse.
